# Effects of Infill Density, Wall Perimeter and Layer Height in Fabricating 3D Printing Products

**DOI:** 10.3390/ma16020695

**Published:** 2023-01-10

**Authors:** Mohammad Azeeb Mazlan, Mohamad Azizi Anas, Nor Aiman Nor Izmin, Abdul Halim Abdullah

**Affiliations:** 1 College of Engineering, School of Mechanical Engineering, Universiti Teknologi MARA, Shah Alam 40450, Selangor, Malaysia; 2 Interdisciplinary Graduate School of Engineering Sciences, Kyushu University, Fukuoka 816-8580, Japan; 3 Biomechanical & Clinical Engineering (BioMeC) Research Group, College of Engineering, Universiti Teknologi MARA, Shah Alam 40450, Selangor, Malaysia

**Keywords:** 3D printing, 3D-printed part, finite element method, tensile testing, Young’s modulus

## Abstract

Three-dimensional printing is widely used in many fields, including engineering, architecture and even medical purposes. The focus of the study is to obtain the ideal weight-to-performance ratio for making a 3D-printed part. The end products of the 3D-printed part are hugely affected by not only the material but also the printing parameters. The printing parameters to be highlighted for this study are the infill density, wall perimeter and layer height, which are the commonly adjusted parameters in 3D printing. The study will be divided into two parts, the simulation analysis and the experimental analysis, to confirm both results toward the trend of Young’s modulus for the material. It will then be analyzed and discussed toward any differences between the two results. The results showed that increasing the value of all three parameters will increase the tensile elasticity of the part.

## 1. Introduction

Three-dimensional printing or additive manufacturing is a type of process to quickly create an actual physical 3D object from a digital 3D model, thus called rapid prototyping [1]. It was thought that 3D printing technology was to be used only for making prototypes, but nowadays, it has been used for different types of applications. It has been used in many fields, including the aerospace industry, automotive industry, food industry, healthcare and medical industry, architecture industry, the fashion industry and the mechanical-electrical industry [2]. The technology was commercialized by Charles Hull in 1980 [3] before becoming widely known in the last decade. The first type of 3D printer that was invented by Charles Hull was of stereolithography (SLA) type. ASTM has categorized 3D printing technologies into seven different types, which are binder jetting, directed energy deposition, material extrusion, material jetting, powder bed fusion, sheet lamination and vat polymerization (SLA category) [4]. Each type has its own pro and cons and is not an all-rounder. One type may suit one application but may not suit another application based on the limitation of the type. The one widely in use is material extrusion or more commonly known as ‘fused deposition modelling (FDM)’. This type uses heated thermoplastic extruded through a small gap or hole along the X–Y axis, layer-by-layer, from bottom to top of the Z axis according to the computer numerical control (CNC) [5]. Due to the quick process of material deposition and hardening thermoplastic on the outcome, it has widely been used not only for prototyping but also for finished or fully functional products [6].

Polylactic acid (PLA) is widely used in many manufacturing or production processes, including injection molding, blow molding and also 3D printing, due to its wide applications in many fields thanks to its biocompatibility, biodegradability, thermal stability and also solvent resistance [7]. It is one type of material commonly used for FDM technology. It is a biodegradable material [8]. PLA was the most common material for FDM due to its good stability during printing [9], but its low melting point due to its glass transition temperature of only 60−65 degrees [10] makes it subject to deformation under sunlight. For the FDM type of 3D printing, the outcome of the 3D-printed part is affected by the printing parameters set up during the pre-printing process. The study of printing parameters will use PLA as the baseline for the type of material used. Other materials, such as acrylonitrile butadiene styrene (ABS) and polyethene terephthalate glycol (PETG), will have different results due to their different mechanical properties, and they were suggested to have a lesser effect on changing parameters [11]. The strength of the 3D-printed part is highly affected by infill density, wall perimeter and layer height [12]. A study shown by J. Maszybrocka suggests that the mechanical properties of a 3D-printed part are highly affected by the core filling (infill) density and also the outer layer (wall perimeter) [13]. The preference of this study is toward making a 3D-printed end product that has an ideal weight-to-performance ratio. The first parameter, which is the infill density percentage, is the density material used for the model; 0% will hollow out the inner part fully, and 100% will totally solidify the inner part. Wall perimeter refers to the outermost layer of the model lying next to each other. An increasing number of wall perimeters will have thicker outermost layers, such as three wall perimeters using a 0.4 nozzle resulting in a 1.2 mm wall perimeter thickness [14]. The wall will act as the foundation of the 3D-printed part and increasing values of wall perimeter would result in a stronger structure. Layer height is the thickness of each layer line deposited before and after each succession of each layer. The layer height is also regarded as printing resolution in the 3D printing community. The lower the layer height values, the higher the printing resolution of the 3D-printed part will be. Five successful layer heights of 0.2 mm will result in 1 mm of print thickness. It is to be noted that different parameters will give different printing times and printing quality [15].

The study will consist of two parts, simulation analysis and experimental analysis, to determine the strength of the 3D-printed part, that is, the Young’s modulus. Young’s modulus is a measurement of a solid’s stiffness under a load, which compares stress to strain along an axis. In simpler terms, it is a measurement of load or stress toward the object when it undergoes elastic deformation until it is unable to return to its original shape after the load is removed [16]. Experimental analyses are needed in order to confirm the accuracy of the simulated data. The testing will follow the American Society for Testing and Materials (ASTM) D638 standards. ASTM D638 will be performed by applying a sample (with a thickness of 1 mm to 14 mm) with a tensile force end to end and measuring the various properties of the specimen under the stress given [17]. Four different tensile properties can be gained from the testing: tensile strength, tensile modulus, elongation and Poisson’s ratio. For simulated analysis, the same sample 3D model needed to run a simulation using CAE software using finite element analysis for a static structure. Both results are to be compared and discussed for their differences, if any differences occur.

The importance of this study is for it to serve as future guidance for making 3D-printed parts or products for PLA material without needing to conduct experimental testing, to save time and resources. Engineers or designers only require making a simulated analysis to gain the best weight-to-performance ratio for the part designed.

## 2. Materials and Methods

The first step of the study is to set up the manipulated parameters with the baseline. The parameters to be adjusted will be infill density, wall perimeter and layer height. The material used will be PLA from Lanbo (LB) with a baseline of 20% infill density, 2 wall perimeter and 0.2 mm layer height. The adjusted value for each parameter will be as follows:

- Infill density (%): 0, 20, 40, 60, 80, 100.
- Wall perimeter: 1, 2, 3.
- Layer height (mm): 0.12, 0.20, 0.28.

The specimen will be coded as in Table 1:

The 3D printer used for the study is Creality Ender 3 V2, and it used these uniform parameters:

- Nozzle temperature: 200 degrees.
- Bed temperature: 60 degrees.
- Base printing speed: 60 mm/s.
- Infill pattern: grid.
- Top/bottom thickness: 0.8 mm.
- Fan Speed: 100%.

**Figure 1:**
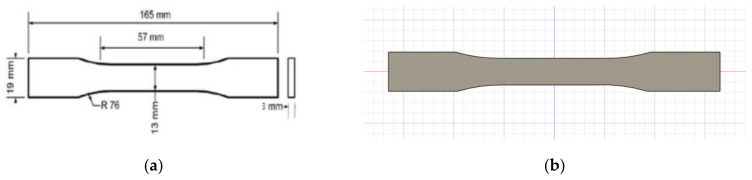
(**a**) Dimensions of ASTM D638 Type I and (**b**) 3D model of sample.

**Figure 2:**
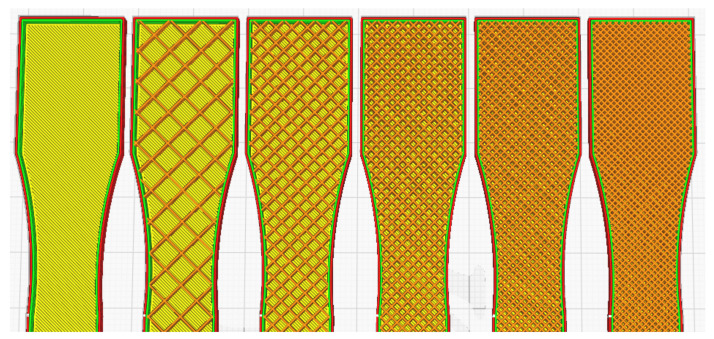
Infill density of sample: 0%, 20%, 40%, 60%, 80% and 100% (left to right).

**Figure 3:**
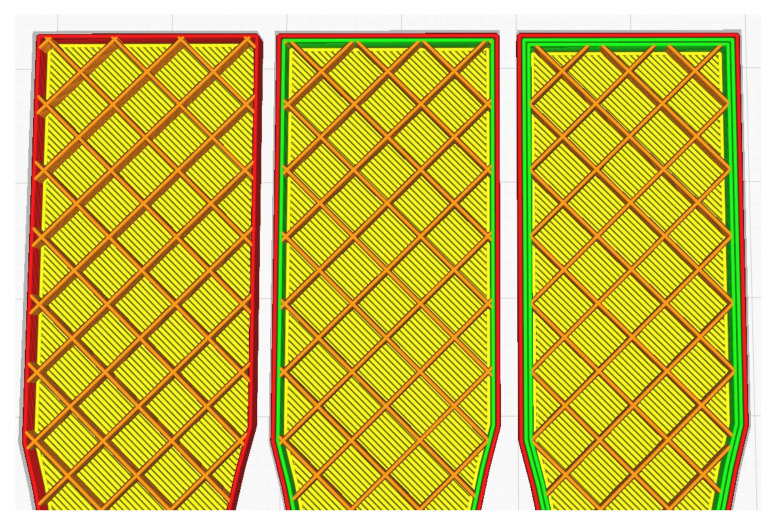
Wall perimeter: 1, 2 and 3 (left to right).

**Figure 4:**
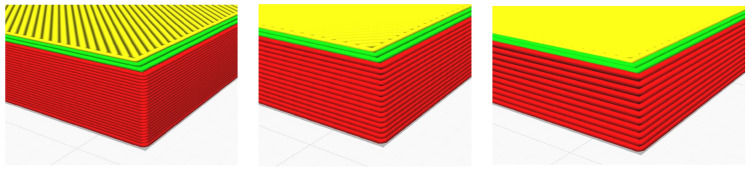
Layer height: 0.12 mm, 0.20 mm and 0.28 mm (left to right).

A 3D model of the testing sample needed to be designed using Autodesk Fusion 360 first, following the standard set by ASTM D638 as per Figure 1. This will be the model used for simulated testing and experimental testing to verify both values. For experimental testing, five (5) identical specimens of each parameter will be tested to obtain the average data, avoiding analytic error. Different types of parameters—infill density, wall perimeter and layer height—are shown in Figure 2, Figure 3 and Figure 4, respectively. Due to current limitations, simulated testing will only be performed for infill density and wall perimeter parameters. This was realized later during the study as software cannot differentiate between different layer heights and bonding between the layers. The slicing software used to set the parameters is Ultimaker Cura.

For simulation analysis, there will only be nine (9) 3D models from two parameters, which are the infill density and wall perimeter designed initially in CAD-CAE software. All nine 3D models will run through a simulation using ANSYS: Workbench 2021 R2 under Static Structural analysis. The data used for the material properties are referred from Table 2. These data were compiled by J. Torres from his studies on bulk PLA material properties. The data were likely to be gathered from averaging between a few brands of PLA tested.

**Figure 5:**
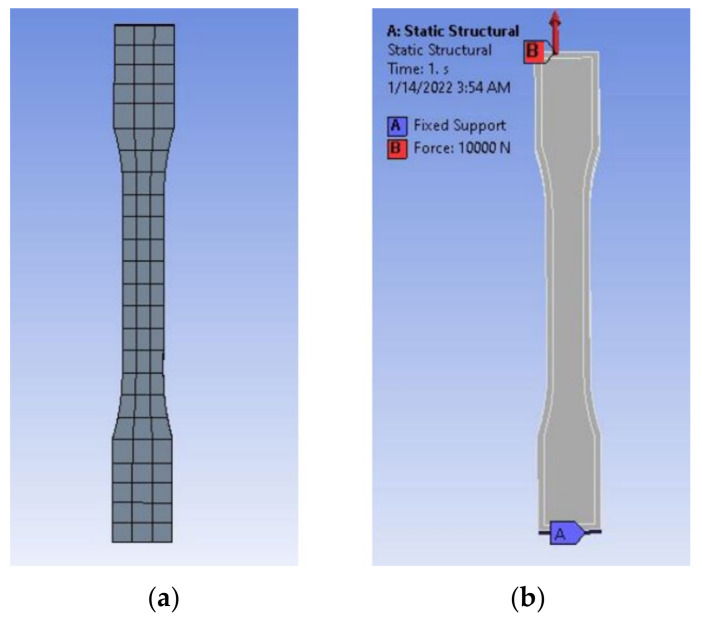
(**a**) Meshing of the specimen and (**b**) boundary and loading conditions of 10 kN.

Once the material property of the 3D model has been set up, the 3D model will need to auto-mesh using the software since the size of the sample is all the same; thus, each model will have approximately the same size set up by the CAE software. In creating a static structural simulation, it is necessary to at least set up fixed support and the force exerted. For this study, a fixed support will be placed at one end while another end will exert a force of 10 kN, as shown in Figure 5.

**Figure 6:**
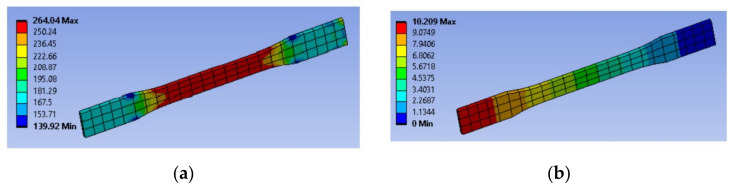
Sample outcomes of the simulation for (**a**) maximum stress (MPa) and (**b**) elongation (mm).

Before running the simulation analysis, a display graph for maximum principal stress and maximum principal elastic strain is shown beside the simulation to obtain the reading before and after the load is exerted. Simulation can be run and repeated for different parameters. Different parameters may lead to longer times for the simulation to complete due to the amount of mesh inside the 3D model. The simulation findings will show the result for max stress and the elongation, as shown in Figure 6 for specimen 6.

From the result, we can obtain the max strain from the elongation and the Young’s modulus from max stress and max strain as per formula:

$$E=\frac{\mathrm{Max}\;\mathrm{Stress}}{\mathrm{Max}\;\mathrm{Strain}}=\frac{\mathrm{Max}\;\mathrm{Stress}}{\Delta L/L}$$

The results are tabulated in Table 3.

**Figure 7:**
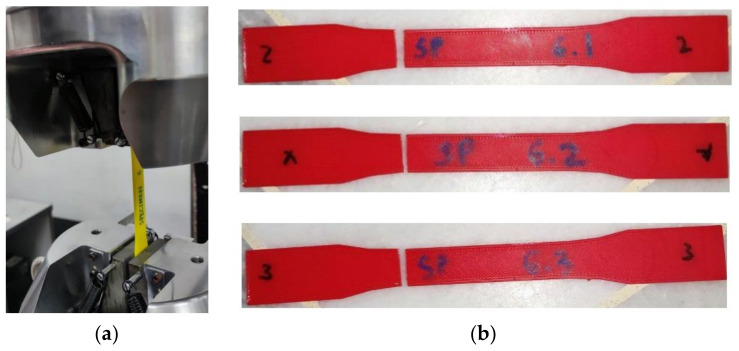
(**a**) Specimen set up in the universal testing machine and (**b**) sample of specimen 6 after testing.

For experimental analysis, the testing was carried out on a universal testing machine (UTM) at the Advanced Strength of Material Laboratory in UiTM Shah Alam, Selangor, Malaysia. The UTM used is the Servopulser Shimadzu with Servo830 for the software to adjust the machine and receive the data. Each specimen was fitted to the UTM and tensile tested at a crosshead setting rate of 5 mm/min until it failed, as shown in Figure 7.

The data from the experimental analysis were calculated and are tabulated in Table 4.

## 3. Results

### 3.1. Different Infill Density Testing

**Figure 8:**
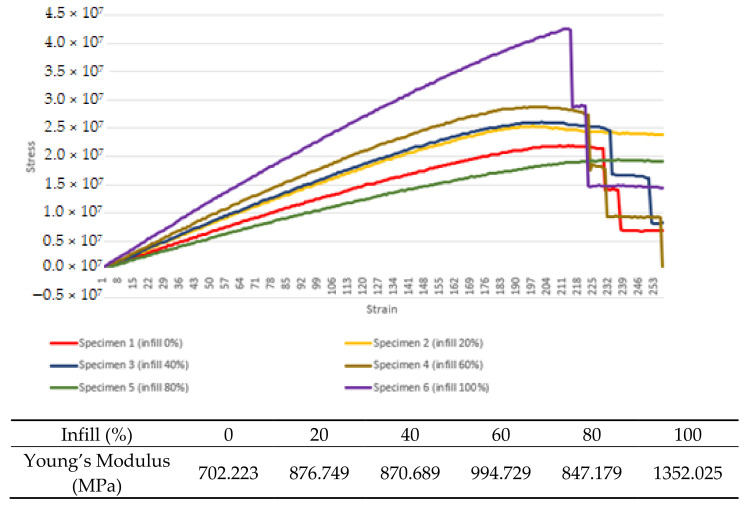
Average experimental stress–strain data for infill density test samples.

Figure 8 shows the comparison between experimental testing of specimens for different infill settings and higher infill results in the ability to withstand a higher load of stress before yielding. The strain is slightly increased for each increment of the infill density. The same behavior is shown when compared to the simulation part of the test. The Young’s modulus values of specimens 1, 2, 3, 4, 5 and 6 are 703.223 MPa, 876.749 MPa, 870.689 MPa, 994.729 MPa, 847.179 MPa and 1352.025 MPa, respectively.

### 3.2. Different Wall Perimeter Testing

**Figure 9:**
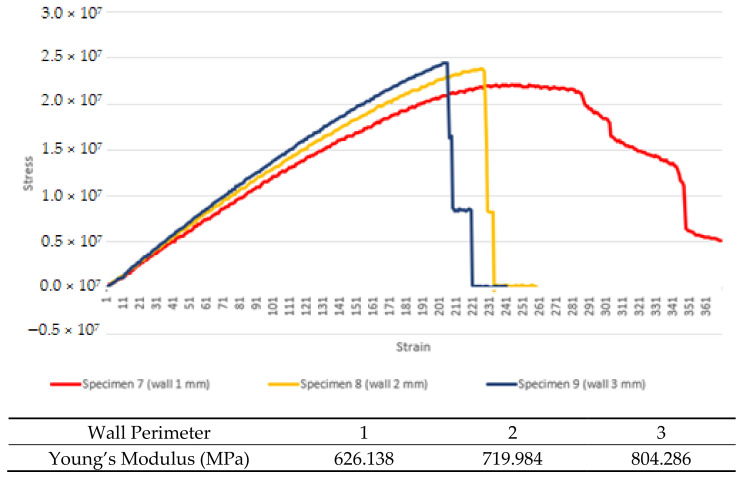
Average experimental stress–strain data for wall perimeter test.

From Figure 9, the max stress of the experimental part is slightly better for higher wall perimeter. This follows the same trends with the simulation parts, even with high differences between the value of the simulated and the experimental parts. The Young’s modulus values of specimens 7, 8 and 9 are 626.138 MPa, 719.984 MPa and 804.286 MPa, respectively.

### 3.3. Different Layer Height Testing

**Figure 10:**
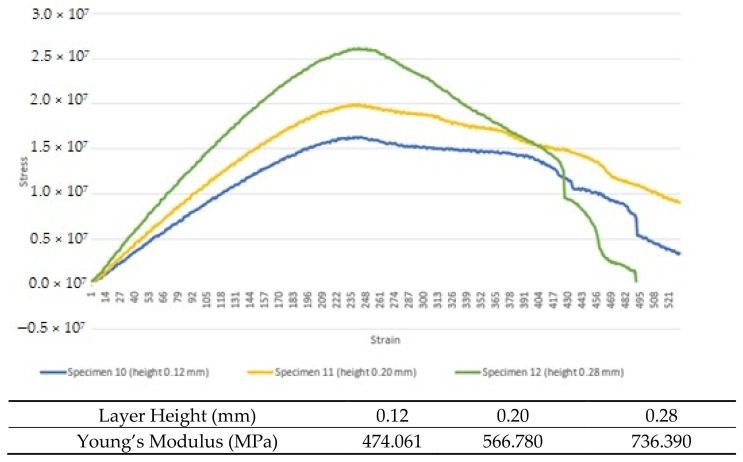
Average experimental stress–strain data for layer height test.

Due to the current limitation of simulation technology to define the difference in layer height structure, no simulated part was performed for the layer height testing. However, experimental testing shows that higher layer height values result in better max stress and thus better a Young’s modulus as in Figure 10. The Young’s modulus values of specimens 10, 11 and 12 are 474.061 MPa, 566.78 MPa and 736.39 MPa, respectively.

### 3.4. Comparison Data for Different Parameter Settings

Both data from the simulation analysis and experimental analysis are compared and discussed based on Table 5. The results were compared to determine the accuracy between the two data. The accuracy of all specimens ranged from 20% to 43%, showing that the simulated data’s Young’s modulus data have a higher value compared to the experimental data.

## 4. Discussion

### 4.1. The Effect of Infill Density in Predicting Young’s Modulus

From the data, it can be concluded that a higher value of infill density will result in better strength and thus a better Young’s modulus value. It can be inferred that with less infill density, the structure or cross-sectional area of the part will be lower, thus resulting in less surface for the structure to hold together when introduced to the load. Some end products may have a weight requirement; thus, making a 100% infill 3D-printed part is not always the right solution. Some parts made for aviation need to be lightweight while maintaining a strong structure.

### 4.2. The Effect of Wall Perimeter in Predicting Young’s Modulus

Both simulated and experimental data show that increasing the wall perimeter has much more effects per increment compared to infill density, as the wall perimeter adds to the rigidity of the outer structure compared to the inner structure for the infill density. The shell or the outer structure is placed beside each perimeter, which have better contact surface for the fusion of plastic compared to the infill, as infill only cross-sectioned the inner part and only fused mostly on the upper and lower layers compared to the perimeter, which fuses from upper, lower and, more importantly, side layers. The critical part of the 3D-printed part is the fusion of deposited filament with the previously deposited filament, unlike the injection-molded part, which lacks the issue.

### 4.3. The Effect of Layer Height in Predicting Young’s Modulus

From the experimental testing, the deduction will be that the one with a higher layer height, and it will have a higher Young’s modulus value. The 3D-printed part usually fails at the fusion of plastic between the layers or due to a delamination issue. With a higher layer height value, there will be fewer layers in the 3D-printed part; thus, there will be fewer failure points in the part. A higher layer weight will have better printing time but will result in a rougher surface. Some 3D-printed parts might have to highlight aesthetic features; thus, printing in a high layer height value will diminish the aesthetic value. Parts or products without such aesthetic requirements should be printed in higher layer height since it would result in better strength and less printing time needed.

### 4.4. The Differences between Simulated and Experimental Analysis

The data show that there are huge differences between simulated analysis and experimental analysis, which can be due to many reasons. One of the main reasons would be the data gained from the literature reviews may not be the same brand as those we had used for experimental testing, even if it was the same type of filament. Different blends and additives may result in drastic differences between simulation and experimental. Another reason may be due to the plastic bonding between the layers not being perfect, thus resulting in a weaker structure when tested in experimental testing compared to simulated analysis, which expects the bonding to be in perfect condition. Any moisture or dust in the filament during printing may also affect the outcome of the print. Future studies should use a filament from a renowned brand such as Polymaker, which has its own testing of material and data sheet for every material they release to be used as a guide for simulation and experimental testing.

From the data shown between the simulated and experimental analysis, we can see that the trend that shows experimental analysis is about 31.67% accurate on average from the simulated analysis. This shows that the 3D-printed part will only be about 31.67% accurate from the simulated data and thus can be used for guidance in making a 3D-printed part. For a safety measure, it can be said that Young’s modulus of the real 3D-printed part will only be 30% of what the simulated analysis shows.

From the data comparing all parameters, we can see that specimen 6 with 100% infill has the highest value of Young’s modulus for both experimental and simulation analyses. For comparing wall perimeters, we can see that an increasing number of wall perimeters will have a higher value of Young’s modulus. This may be due to an increase in wall perimeter adding to the rigidity of the whole structure the same as the infill density parameters. For the layer height, the sample was only tested for experimental analysis due to the current limitation of CAE software. Increasing the layer height of the specimen will add to a better value of Young’s modulus. This may be due to lowering the layer height of the specimen having more layers in total for the specimen, thus allowing for more chances of failure at the plastic bonding of each layer. The higher layer height of 3D printing parameters will have lower layers in total, subsequently lowering the chances of plastic bonding failure.

## 5. Conclusions

The study was first carried out to confirm that the value of the simulated analysis is accurate with the experimental analysis values, but the value turned out to be very different but with a trend of being about 22.82% accurate from the simulated data. This may be due to many reasons, such as materials, dust, moisture or even human error. However, with the trend of being 22.82% accurate, future studies that follow may be about 20% accurate from the simulated value to the real 3D-printed part. From the study of parameters, we know that increasing the infill values, increasing the wall perimeters and increasing the layer height would increase the value of Young’s modulus. However, increasing these values will also increase the weight of the printed part. With these data being known, one can fabricate a functional 3D-printed part toward an ideal weight-to-performance ratio. Twenty percent of the simulated value data must be compared with the real testing data; otherwise, the safety factor of the 3D-printed part will not be obtained. This conclusion should be true with other types of 3D-printed material, especially for ABS or PETG, which should have slightly different mechanical properties but will stay true to the conclusion.

## Figures and Tables

**Table 1:** Coded specimen according to parameters (darkened section is fixed parameter).

	Infill Density (%)	Wall Perimeter	Layer Height (mm)
Specimen 1	0	2	0.20
Specimen 2	20	2	0.20
Specimen 3	40	2	0.20
Specimen 4	60	2	0.20
Specimen 5	80	2	0.20
Specimen 6	100	2	0.20
Specimen 7	20	1	0.20
Specimen 8	20	2	0.20
Specimen 9	20	3	0.20
Specimen 10	20	2	0.12
Specimen 11	20	2	0.20
Specimen 12	20	2	0.28

**Table 2:** PLA material properties [18].

Mechanical Property of PLA	
Density (ρ)	1.24 g/cm^3^
Elastic modulus (E)	3500 MPa
Shear modulus (G)	1287 MPa
Poisson’s ratio (v)	0.36
Yield strength (σ_y_)	70 MPa
Ultimate tensile strength (S_ut_)	73 MPa
Elongation	~7%

**Table 3:** Results for the simulation testing.

	Infill Density (%)	Wall Perimeter	Layer Height (mm)	Max Stress (MPa)	Elongation (mm)	Young’s Modulus (MPa)
Specimen 1	0	2	0.20	264.04	26.842	1623.076
Specimen 2	20	2	0.20	427.48	28.132	2507.259
Specimen 3	40	2	0.20	489.84	30.131	2682.407
Specimen 4	60	2	0.20	565.43	32.321	2886.543
Specimen 5	80	2	0.20	642.24	35.348	2997.895
Specimen 6	100	2	0.20	792.77	35.348	3700.550
Specimen 7	20	1	0.20	396.63	26.444	2474.813
Specimen 8	20	2	0.20	427.48	28.132	2507.259
Specimen 9	20	3	0.20	834.37	35.898	3835.062
Specimen 10	20	2	0.12	-	-	-
Specimen 11	20	2	0.20	-	-	-
Specimen 12	20	2	0.28	-	-	-

**Table 4:** Results for the experimental testing.

	Infill Density (%)	Wall Perimeter	Layer Height (mm)	Young’s Modulus (MPa)
Specimen 1	0	2	0.20	703.22
Specimen 2	20	2	0.20	876.75
Specimen 3	40	2	0.20	870.69
Specimen 4	60	2	0.20	994.73
Specimen 5	80	2	0.20	847.18
Specimen 6	100	2	0.20	1352.03
Specimen 7	20	1	0.20	626.14
Specimen 8	20	2	0.20	719.99
Specimen 9	20	3	0.20	804.29
Specimen 10	20	2	0.12	474.061
Specimen 11	20	2	0.20	566.78
Specimen 12	20	2	0.28	739.36

**Table 5:** Comparison between simulated analysis and experimental analysis.

	Young’s Modulus (MPa) from Simulation Analysis	Young’s Modulus (MPa) from Experimental Analysis	$\mathrm{Accuracy}\;(\%)=\;\left(\frac{Experimental}{Simulation}\right)\;\times \;100$
Specimen 1	1623.076	703.22	43.33
Specimen 2	2507.259	876.75	34.97
Specimen 3	2682.407	870.69	32.46
Specimen 4	2886.543	994.73	34.46
Specimen 5	2997.895	847.18	28.26
Specimen 6	3700.55	1352.03	36.54
Specimen 7	2474.813	626.14	25.30
Specimen 8	2507.259	719.99	28.72
Specimen 9	3835.062	804.29	20.97
Specimen 10	-	474.061	-
Specimen 11	-	566.78	-
Specimen 12	-	739.36	-

## Data Availability

The data presented in this study are available on request from the corresponding author.
